# Flexible work, rigid politics: Platformization, aspirations, and the making of the authoritariat in the Global South

**DOI:** 10.1177/29768640251379552

**Published:** 2025-09-23

**Authors:** Rosana Pinheiro-Machado, Miguel Paolo Rivera, Wagner Guilherme Alves da Silva, Rashmi Guha Ray, Marina Frid, Jessica Matheus de Souza

**Affiliations:** 8797 University College Dublin; 8797 University College Dublin; Ateneo de Manila University; 8797 University College Dublin; 8797 University College Dublin; 8797 University College Dublin; 8797 University College Dublin

**Keywords:** Brazil, India, Philippines, platformization, authoritarian populism

## Abstract

This article examines the intersection of flexible digital labor and rigid authoritarian politics in the Global South. Based on ethnographies in Brazil, India, and the Philippines, it introduces the concept of the authoritariat to describe segments of the working class drawn to reactionary populism through a combination of economic precarity and aspirational desire. Major digital labor platforms, as ideological technologies, enable a bifurcation that recenters the individual and fosters a perceived detachment from politics, thereby deepening the abyss between labor and political life. As platform work and digital entrepreneurship reshape notions of success, autonomy, and class identity, many workers come to embrace authoritarian leaders who promise order, moral certainty, and opportunity. The article argues that the emotional and economic logic of the digital economy play a key role in transforming political subjectivity. Far from passive victims or ideologically radicalized citizens, these individuals navigate a contradictory terrain of exclusion and self-worth.

Contemporary scholarship on the rise of authoritarian populism among working-class sectors tends to focus predominantly on reactive and negative emotions, such as anger, fear, hatred, and resentment (e.g., Miller-Idriss, 2022). Accordingly, digital platforms are often depicted as vehicles that fuel such emotions through misinformation. While anger and misinformation are undoubtedly central to illiberal politics on social media, they provide a limited understanding of the political phenomenon. This article argues that the growing platformization of precarious workers—encompassing both work applications and social media—profoundly impacts their political subjectivities, shaping anger but also—perhaps more importantly—capturing dreams. We contend that a full understanding of what binds people to illiberal regimes must include the active and positive emotional appeals cultivated and amplified by platforms.

Drawing on multi-sited ethnographic research on digital labor and platform labor across various precarious sectors in Brazil, India, and the Philippines, we examine the intersection between platforms and illiberalism. We explore how this nexus mobilizes the active and positive emotions of what we call the *authoritariat*, a new working class that is increasingly platformed and inclined toward illiberalism in the Global South. The existence of a reactionary working class is a phenomenon as old as capitalism itself (e.g., Marx, 1963), presently being profoundly transformed by platformization. Authoritarian populism has harnessed segments of marginalized groups by appealing to their aspirations for recognition, autonomy, material comfort, and freedom. Platforms then amplify these positive emotions. Our argument is that major digital labor platforms, as ideological technologies, enable a bifurcation that recenters the worker and fosters a perceived detachment from politics, thereby deepening the abyss between labor and political life. Individuals are portrayed as solely responsible for their economic success, which is framed as a matter of personal merit, while the role of the state is seen as minimal, required to be only a “strongman” that enables deregulation and selective interventions in society.

The authoritariat largely belongs to a demographic group that has undergone historical processes of marginalization and subalternation in now emerging economies marked by a colonial past, persistent authoritarianism, and unstable democratic institutions. Now, in precarious and flexible employment, these masses seek to carve out space in society and shape their own narratives. Unlike in the Global North, these are not white working-class people, nor are they the “left behind” victims of deindustrialization. In the Global South, we are witnessing the uncertain rise of those who experienced limited upward mobility during the economic growth of emerging markets—amidst major national narratives of hope. This is why we argue that frameworks rooted in negative emotions, often used to explain far-right support in the Global North, are insufficient for understanding support for authoritarian politics in the Global South. Here, we are dealing with aspirational masses, motivated by religion, entrepreneurialism, national security, and transnational mobility, among others.

The authoritariat navigate lives marked by structural inequalities and liminal class conditions, which explains their constant struggles between negative and positive political emotions. These groups have become new consumers, heavily indebted, and rebranded as entrepreneurial subjects through aspirational narratives or platform-based promises of flexibility and opportunity. They are no longer classified as the “urban poor,” but they are not yet fully “middle class” (Pertierra et al., 2025). They experience rage at the grind of everyday life, fear of losing what little they have gained, and resentment for what they have not accomplished. However, they continue to nurture dreams of autonomy, freedom, dignity, recognition, and social mobility within societies that have long denied them belonging—aspirations now increasingly enabled by digital technologies. These enable individuals to reimagine and remake themselves and to emphasize personal transformation.

At present, there is significant evidence demonstrating that these demographic majorities are strongly aligned with illiberal populism in Brazil, India, and the Philippines (e.g., Barlach and Mendes, 2022; Jaffrelot, 2015; Lero, 2023). To understand this political inclination, five researchers have conducted simultaneous long-term, on-the-ground ethnographies in communities of precarious workers in these countries, combined with digital ethnography, and have carried out 101 in-depth interviews across labor sectors in large cities. These sectors include cleaning, retail, beauty and wellness, app-based drivers and riders, content creators, etc. Across all field sites, we consistently found that aspirations play a central role in cultivating political subjectivities.

It is well-documented that many workers come to see themselves as strivers solely responsible for their own destinies—embracing an “every person for themselves” ethos—and regard the role of the state as minimal. Their ambivalence towards the state's capability to make an economic difference in their lives coexists with a desire for a rigid strongman leader perceived as capable of dealing with “bad people”—a category that may include drug dealers, Muslims, or other internally constructed enemies—and, if necessary, dismantling state power itself. The intersection between labor precariousness and illiberalism is enabled by a paradox we name in the title of this article: *flexible work, rigid politics*.

Here, labor platforms play a central role in allowing individuals to work under a perceived autonomy, leading to the maximization of individual effort through endless self-competition—an endeavor that ultimately becomes a matter of faith. Across the three sites, platform workers perceived the flexibility to manage personal and working hours as a genuine benefit and as a new way to gain control of their lives. For example, for platform riders and drivers in these countries who would historically have been destined for intensive labor regimes in low-paying and unrewarding jobs, the possibility of riding or driving everywhere represents an allegory of freedom of movement: individuals choose when to start a shift, when to pause for a meal, and when to stop (see Pinheiro-Machado and Neves, 2025).

In interviews, Uber drivers and riders in Bangalore articulated a clear vision of the state's role: it should not regulate the economy but simply allow them to work. From this perspective, Prime Minister Narendra Modi was seen as “doing a good job” by fostering an entrepreneurial culture and refraining from interfering in people's economic lives. Numerous food delivery riders also expressed their admiration for strong leaders since they ensure local and national security. In the Philippines, delivery riders expressed almost the same sentiment: that the government's role in their work primarily involves keeping streets free from crime and thus making them safer to traverse, especially at night. Ex-President Rodrigo Duterte's bloody drug war was perceived by many, especially by riders, as having created these “safe and crime-free” environments despite being aware that this safety came at the cost of disproportionate terrorizing of poor urban communities. Most Filipinos, therefore, supported Duterte's drug war but were opposed to the violations of human rights perpetrated by the regime (Presto and Curato, 2024).

The aspiration for freedom finds fertile ground in illiberal contexts marked by neoliberalism. While precarious workers’ yearning for freedom could be interpreted as an expression of neoliberal rationality as defined by Dardot and Laval (2014), such explanation lacks historical contextualization (Pinheiro-Machado and Neves, 2025). In countries like Brazil, India, and the Philippines, large populations, ranging from 30% to 80% of the working population (ILO, 2025), have historically remained a part of the informal economy. As our research suggests, the state has long been perceived as dysfunctional, elitist, corrupt, absent, or violent—or all of these at once. Politics, by extension, is seen as deeply cynical and clientelist, where people have learned to seek survival and enjoyment despite structural constraints. Illiberal populists capitalize on this long-standing anti-system sentiment, cynically portraying themselves as outsiders.

When the state has historically been regarded as inept, and jobs have primarily served to reproduce subaltern, near-enslavement conditions, freedom—perceived as the flexibility of time coupled with the desire not to have a boss—becomes a much-sought aspiration among those who viewed the state as corrupt long before the rise of contemporary populist, anti-establishment appeals. Yet, this autonomy is contradictory, as workers endure long, self-exploitative hours under a gamified algorithmic management (Queiroz et al., 2021). At the same time, our ethnographic stance takes seriously what our interlocutors say: that they work tirelessly but—unlike in other jobs—they can stop working whenever they want. This, fundamentally, represents the feeling of control over their own lives—a feeling that no other conventional wage-labor position has been able to offer. Most of the time, bosses represent humiliation, subordination, and rigidity. As a rider from Metro Manila elaborates: “no toxic workmates or bosses, no HR department, no asking for leaves or breaks.”

Our research suggests that similar emotions drive those pursuing digital entrepreneurship on social media in Brazil.^1^ At the core of the phenomenon is a desire for recognition, respectability, and self-worth, which drives the growing use of Instagram as a tool for promoting businesses, selling products or services online, and engaging in influencer practices. This process is not merely an economic phenomenon; it is also political, as the field of digital marketing on Instagram is shaped by an ecosystem of influencers who establish norms, rules, and mentoring practices that are followed by millions. Our analysis of top influencers (over one million followers) shows that 87% align with Brazil's far right. As this ecosystem comes to monopolize entrepreneurial know-how on Instagram, the dream of making money online increasingly intersects with a network of right-leaning influencers.

Among beauty workers in Brazil, we observed that engaging with social media gives them a sense of legitimacy as “serious” and skilled professionals and a career path that goes from serving individual clients to becoming instructors and mentors of other workers. Their ultimate dream is to achieve fame in the beauty industry and earn the autonomy to pace their work. Similarly, domestic workers seeking to gain a foothold on social media reframe the precarity experienced throughout their lives as a narrative that brands them as “entrepreneurs” rather than “domestic workers.” By rejecting the label of domestic workers, they perceive themselves as having the power to invert the established power dynamics in their line of work.

The impact of this phenomenon is profound. Here, the role of influencers and social media is alarming they sell dreams, values, and lifestyles disguised as “non-political,” to marginalized groups. Although investment in digital businesses may lead to erratic financial returns or other failures, they offer a sense of respectability, the perceived softening of hierarchies, and the possibility of a life of material comfort. This has an enormous impact on the subjectivity and personhood-making of individuals who have long suffered discrimination, poverty, scarcity, and who have been made to feel like “nobodies.”

In sum, we argue that platformization fosters illiberalism for two key reasons: first, by detaching labor from politics through individualizing and deregulated technologies that enable flexibility; and second, by capturing the imaginations of those whose lives are shaped by economic precarity and discrimination. Across the cases presented here on platform labor and social media entrepreneurship, autonomy, anti-hierarchy, and recognition emerge as central aspirations of the authoritariat. Authoritarian populism has managed to craft a narrative that fosters self-worth and a sense of deserving. Platformization, in this context, appears as a perceived means of attaining freedom in the face of persistent processes of subalternation.

## Notes

1. For a detailed view on this theme, see our project's report on labor market on Instagram: Pinheiro-Machado, R., Matheus, J., Frid, M., Silva-Alves, W., Petra, P., and Penalva, J. (2024). Social Media as a Digital Labour Platform: Assessing the Social, Cultural, and Political Impacts of Labour Market Migration to Instagram. Digital Economy and Extreme Politics Lab, N. 2024.1.
